# Cardiovascular protection by SGLT2 inhibitors: an integrative review of mechanistic networks, clinical evidence, and safety considerations

**DOI:** 10.3389/fcvm.2026.1776906

**Published:** 2026-06-09

**Authors:** Ziyun Wang, Yijin Wang, Wangning Shangguan, Tao Li

**Affiliations:** 1 Department of Anesthesiology, The Affiliated Hospital, Southwest Medical University, Luzhou, Sichuan, China; 2 Department of Anesthesiology and Perioperative Medicine, The Second Affiliated Hospital and Yuying Children’s Hospital of Wenzhou Medical University, Wenzhou, China; 3 Department of Anesthesiology, Laboratory of Mitochondria and Metabolism, National-Local Joint Engineering Research Centre of Translational Medicine of Anesthesiology, West China Hospital, Sichuan University, Chengdu, Sichuan, China

**Keywords:** cardiovascular diseases, dapagliflozin, empagliflozin, heart failure, sodium-glucose cotransporter 2 inhibitors

## Abstract

Sodium-glucose cotransporter 2 (SGLT2) inhibitors were originally developed as glucose-lowering agents for type 2 diabetes mellitus, but accumulating evidence has demonstrated their broad cardiovascular and cardiorenal benefits beyond glycemic control. In particular, empagliflozin and dapagliflozin have shown robust prognostic benefits in heart failure with reduced ejection fraction (HFrEF) and expanding therapeutic value in heart failure with preserved ejection fraction (HFpEF). However, their cardiovascular protection cannot be fully explained by glucose lowering alone and is likely mediated through a multilayered network of systemic, metabolic, vascular, and cellular mechanisms. This integrative narrative review summarizes current mechanistic and clinical evidence regarding SGLT2 inhibitors in cardiovascular disease and proposes a translational framework linking hemodynamic rebalancing, cardiometabolic reprogramming, vascular protection, redox homeostasis, mitochondrial quality control, immunometabolic modulation, and attenuation of adverse myocardial remodeling. Clinically, the strongest evidence has been established in heart failure, whereas data in ischemic heart disease, post-myocardial infarction remodeling, and arrhythmias remain promising but less definitive. In post-myocardial infarction settings, SGLT2 inhibitors may improve N-terminal pro-B-type natriuretic peptide (NT-proBNP) levels, ventricular remodeling parameters, and heart failure-related outcomes, although effects on hard clinical endpoints remain inconsistent. For atrial arrhythmias, emerging evidence suggests a potential reduction in atrial fibrillation or flutter risk, but dedicated trials are still required. Safety considerations, including genital infections, volume depletion, renal function changes, euglycemic ketoacidosis, and perioperative management, should also be incorporated into individualized clinical decision-making. Overall, SGLT2 inhibitors should be viewed as cardiovascular-metabolic modulators acting through interconnected mechanisms, with future research needed to clarify phenotype-specific benefits and optimize their precision use in cardiovascular disease.

## 1 Introduction

Cardiovascular disease (CVD) is one of the leading global health problems and continues to impose a substantial disease burden. As of 2023, CVD accounted for approximately 437 million disability-adjusted life years (DALYs) and 19.2 million deaths worldwide (1). Although many countries have achieved reductions in age-standardized CVD mortality through improvements in tobacco control, blood pressure management, lipid-lowering therapy, and acute cardiovascular care, population growth, population aging, and the increasing prevalence of metabolic risk factors, including obesity, diabetes, hypertension, and elevated Low-Density Lipoprotein Cholesterol (LDL-C), continue to drive the rising absolute burden of CVD (1, 2). Consequently, the numbers of incident cases, prevalent cases, and deaths from CVD are projected to continue increasing in the future (3). In terms of disease composition, ischemic heart disease and stroke remain the leading contributors to global CVD deaths and DALYs (4). Overall, the global epidemiological pattern of CVD is characterized by declining age-standardized rates but increasing absolute burden, with substantial regional disparities. High-income countries generally have lower age-standardized mortality rates, but continue to experience a considerable chronic disease burden due to population aging and the growing number of long-term survivors with CVD. In contrast, Eastern Europe, Central Asia, South Asia, and parts of sub-Saharan Africa still face high rates of premature mortality and disability burden attributable to CVD (5–7).

Given this multidimensional challenge, it is imperative to intensify efforts in public health education, promote healthier lifestyle choices, and optimize healthcare systems to enhance the prevention and management of CVD. These measures are crucial to mitigating the growing health and economic burden posed by cardiovascular disease worldwide.

The sodium-glucose cotransporter (SGLT) family plays a central role in renal glucose reabsorption. Under normal physiological conditions, glucose filtered by the glomerulus enters the renal tubules and is reabsorbed primarily by two transporters: SGLT1 and SGLT2. Among them, SGLT2 is predominantly expressed in the S1 segment of the proximal tubule, where it is responsible for approximately 90% of glucose reabsorption. In contrast, SGLT1 functions as a low-capacity transporter and is mainly active in the kidney and gastrointestinal tract (8, 9). Building upon this physiological mechanism, sodium-glucose cotransporter 2 (SGLT2) inhibitors have emerged as a novel class of oral antidiabetic agents. These drugs lower blood glucose levels by inhibiting glucose reabsorption in the proximal tubules, thereby increasing urinary glucose excretion (10). The efficacy of SGLT2 inhibitors is closely linked to the degree of hyperglycemia and the level of renal function. Currently, the U.S. Food and Drug Administration (FDA) and regulatory authorities in the European Union and other countries have approved several SGLT - 2 inhibitors, including canagliflozin (Invokana), dapagliflozin (Farxiga), empagliflozin (Jardiance), ertugliflozin (Steglatro), and bexagliflozin (Brenzavvy) (11). These agents share similar pharmacokinetic profiles, characterized by rapid oral absorption, a long elimination half-life that permits once-daily dosing, extensive hepatic metabolism primarily via glucuronidation into inactive metabolites, and minimal renal excretion of the parent compound. However, it is important to note that the therapeutic efficacy of SGLT2 inhibitors declines with progressive renal impairment. Consequently, SGLT2 inhibitors are used in patients with an estimated glomerular filtration rate (eGFR) of ≥60 mL/min/1.73 m^2^ for dapagliflozin, or ≥45 mL/min/1.73 m^2^ for canagliflozin, empagliflozin, and most likely ipragliflozin due to attenuated therapeutic effects and potential safety concerns (12, 13).

Beyond their glucose-lowering effects, SGLT2 inhibitors have demonstrated significant cardiovascular benefits in multiple large-scale clinical trials. These cardioprotective effects have been observed not only in patients with type 2 diabetes mellitus(T2DM) but also in non-diabetic populations (14, 15). These findings suggest that SGLT2 inhibitors hold therapeutic value beyond glycemic control and may represent a novel strategy for the prevention and management of cardiovascular diseases.

In light of these developments, the cardiovascular benefits of SGLT2 inhibitors should not be interpreted solely as a consequence of glucose lowering, nor as the result of isolated mechanisms. Instead, accumulating evidence suggests that their cardioprotective effects arise from a multilayered network involving systemic hemodynamic regulation, cardiometabolic adaptation, vascular protection, redox homeostasis, mitochondrial quality control, immunometabolic modulation, and attenuation of adverse myocardial remodeling. Therefore, this integrative narrative review aims to synthesize current mechanistic and clinical evidence on SGLT2 inhibitors in CVD and to propose a translational framework linking molecular mechanisms with distinct cardiovascular phenotypes. By clarifying the interplay and potential hierarchy of these mechanisms, this review seeks to provide a more coherent understanding of SGLT2 inhibitor–mediated cardiovascular protection and to inform their optimized application in clinical practice.

## 2 Literature search strategy and review scope

This article is an integrative narrative review of the mechanistic, translational, and clinical evidence regarding SGLT2 inhibitors in cardiovascular disease. Relevant studies published up to May 2026 were identified through searches of PubMed, Web of Science, Google Scholar, and major guideline documents. Search terms included “SGLT2 inhibitors,” “dapagliflozin,” “empagliflozin,” “canagliflozin,” “heart failure,” “HFrEF,” “HFpEF,” “ischemic heart disease,” “myocardial infarction,” “atrial fibrillation,” “arrhythmia,” “endothelial function,” “oxidative stress,” “mitochondrial function,” “inflammation,” “myocardial remodeling,” “adverse effects,” and “perioperative management.”

Randomized controlled trials, cardiovascular outcome trials, meta-analyses, clinical guidelines, observational studies, and mechanistic preclinical studies were considered. Evidence was selected and synthesized according to its relevance to cardiovascular protection, mechanistic plausibility, clinical impact, and contribution to an integrated framework. As this was not a systematic review or meta-analysis, formal risk-of-bias assessment and quantitative pooling were not performed.

## 3 Integrated mechanisms of SGLT2 inhibitor in cardiovascular protection: From hemodynamic rebalancing to myocardial remodeling

### 3.1 Hemodynamic rebalancing: osmotic diuresis, natriuresis, and ventricular load reduction

After glomerular filtration, filtered glucose is almost completely reabsorbed in the proximal renal tubules, with SGLT2 located on the brush-border membrane of the S1/S2 segments accounting for the majority of glucose reabsorption. By selectively inhibiting SGLT2 in the proximal tubule, SGLT2 inhibitors reduce sodium-glucose cotransport, increase urinary glucose excretion, and enhance distal sodium delivery. This glucose-lowering mechanism is independent of insulin secretion and insulin-mediated peripheral glucose uptake. By increasing urinary glucose excretion and distal sodium delivery, SGLT2 inhibitors induce mild osmotic diuresis and an early natriuretic effect (16–18). Unlike conventional loop diuretics, the diuretic action of SGLT2 inhibitors is generally modest and may preferentially reduce interstitial fluid rather than intravascular volume (19). This property may help relieve congestion while limiting excessive plasma volume depletion and hypoperfusion. These mechanisms provide an important basis for the ability of SGLT2 inhibitors to reduce cardiac volume load and improve hemodynamic status in heart failure. With regard to blood pressure regulation, SGLT2 inhibitors usually produce a gradual and modest antihypertensive effect. Previous studies have shown that SGLT2 inhibitors reduce systolic blood pressure by approximately 3–6 mmHg and diastolic blood pressure by approximately 1–1.5 mmHg (20). A meta-analysis including 10 studies and 2,747 patients with T2DM and hypertension further demonstrated that SGLT2 inhibitors significantly reduced 24-hour ambulatory systolic and diastolic blood pressure, suggesting that their blood pressure-lowering effect is sustained and clinically meaningful (21). Beyond regulating volume status and blood pressure, SGLT2 inhibitors may also directly improve cardiac filling pressures and pulmonary hemodynamics. In an exploratory study involving patients with T2DM and established or high-risk cardiovascular disease, right heart catheterization and norepinephrine spillover kinetics were used to evaluate the hemodynamic and sympathetic effects of empagliflozin. The study found that 12 weeks of empagliflozin treatment lowered pulmonary capillary wedge pressure without producing the anticipated reflex increase in sympathetic nervous activity. This suggests that SGLT2 inhibitors may reduce cardiac filling pressures while avoiding marked sympathetic overactivation (22).

Overall, SGLT2 inhibitors improve volume status and reduce extracellular fluid overload by promoting glycosuria, natriuresis, and mild osmotic diuresis. In parallel, their modest blood pressure-lowering effect and potential improvement in arterial stiffness may further reduce cardiac preload and, to some extent, afterload, thereby decreasing ventricular wall stress, improving ventricular filling pressures, and facilitating favorable ventricular remodeling. These hemodynamic effects represent an important mechanism by which SGLT2 inhibitors improve heart failure outcomes, particularly the early reduction in heart failure hospitalization observed in clinical trials. However, the cardiovascular benefits of SGLT2 inhibitors cannot be fully explained by volume unloading and blood pressure reduction alone. They may also involve deeper metabolic adaptations, including remodeling of energy substrate utilization, improvement in myocardial metabolic efficiency, and regulation of fatty acid oxidation and ketone body metabolism. Therefore, beyond hemodynamic unloading, metabolic reprogramming may constitute another key mechanism underlying the sustained cardioprotective effects of SGLT2 inhibitors.

### 3.2 Cardiometabolic reprogramming: metabolic burden reduction, epicardial Fat remodeling, and myocardial energy optimization

#### 3.2.1 Glycosuria-Induced caloric loss and weight reduction

The weight-reducing effect of SGLT2 inhibitors is primarily attributed to their inhibition of glucose reabsorption in the renal proximal tubules. This mechanism does not depend on insulin secretion or insulin-mediated peripheral glucose uptake (18). Following SGLT2 inhibition, urinary glucose excretion increases, resulting in a sustained caloric loss, with approximately 200–250 kcal of energy lost in the urine per day (23). In addition, glycosuria can induce mild osmotic diuresis, thereby promoting fluid loss and contributing to the early reduction in body weight during treatment (24). With prolonged treatment, SGLT2 inhibitor-associated weight loss is more closely reflected by reductions in fat mass, including decreases in both visceral and subcutaneous adipose tissue (25, 26). Moreover, SGLT2 inhibitors may promote a shift in substrate utilization from predominant glucose oxidation toward increased fatty acid oxidation and ketone body production, thereby further contributing to fat mass reduction and body weight control (27).

#### 3.2.2 Uric acid lowering and metabolic stress attenuation

SGLT2 inhibitors have also been shown to lower serum uric acid concentrations by promoting renal uric acid excretion, thereby contributing to an improved overall metabolic profile (28). A recent meta-analysis revealed that SGLT2 inhibitors significantly reduce serum uric acid levels in patients with severe chronic kidney disease (CKD) (29). Similarly, effective treatment with empagliflozin has been associated with substantial reductions in serum uric acid levels among patients with diabetes (30). Although hyperuricemia has been proposed to cause cell apoptosis and endothelial dysfunction through mechanisms such as inflammasome activation and oxidative stress, the causal relationship between high serum uric acid levels and other metabolic or cardiovascular diseases remains controversial (31, 32). Nevertheless, reducing serum uric acid levels may help improve the metabolic milieu in patients with cardiovascular diseases and mitigate potential cardiovascular risk factors.

#### 3.2.3 Epicardial adipose fat remodeling and local cardiac microenvironment

Epicardial adipose tissue (EAT) is a type of visceral fat located between the visceral pericardium and the myocardium, encompassing the perivascular adipose tissue surrounding the coronary arteries. EAT possesses endocrine functions and can secrete various bioactive factors into the systemic circulation, thereby influencing cardiovascular physiology (33). Increasing evidence suggests that EAT plays a pathogenic role in the development of several cardiovascular diseases, including coronary artery disease (CAD), heart failure (HF), and arrhythmias (34).

Emerging evidence indicates that treatment with dapagliflozin for six months not only significantly improves glycemic control but also markedly reduces the volume of EAT. Moreover, changes in EAT volume are strongly correlated with changes in body weight and tumor necrosis factor-alpha (TNF-α) levels. These findings suggest that dapagliflozin may exert cardiovascular protective effects not only through systemic metabolic improvements—including reductions in plasminogen activator inhibitor-1 (PAI-1) and TNF-α—but also via local modulation of EAT volume (34).

Further supporting this notion, a separate study demonstrated that four weeks of dapagliflozin treatment significantly decreased both EAT thickness and EAT glucose uptake in patients with T2DM and stable CAD—by 19% and 21.6%, respectively. Notably, the reduction in EAT FDG uptake was paralleled by an improvement in myocardial blood flow reserve, suggesting a potential mechanistic link between EAT metabolic activity and myocardial perfusion. These findings imply that dapagliflozin may improve cardiac function and protect the cardiovascular system by regulating the metabolism and inflammatory profile of EAT (35).

#### 3.2.4 Substrate flexibility, ketone utilization, and myocardial energy optimization

In cardiomyocytes, mitochondria occupy approximately one-third of the cell volume and are responsible for producing 95% of Adenosine Triphosphate (ATP), primarily through the oxidative phosphorylation (OXPHOS) pathway (36). The heart exhibits remarkable metabolic flexibility, allowing it to efficiently utilize a variety of energy substrates—including fatty acids, glucose, lactate, and ketone bodies—depending on its ATP demands (37). However, under chronic pathological conditions such as HF and T2DM, cardiac metabolic pathways become dysregulated, contributing to structural and functional abnormalities in the heart (38, 39).

Ketone bodies have emerged as important alternative energy substrates in the failing heart. As mitochondrial fuels, ketone bodies can be converted into acetyl-CoA and enter the tricarboxylic acid cycle, thereby supporting ATP production under conditions of metabolic stress (40). Experimental studies suggest that SGLT2 inhibitors may enhance ketone body availability and utilization, thereby improving myocardial energy efficiency (41). In models of diabetic cardiomyopathy, empagliflozin has been shown to promote ketone body synthesis by increasing the activity of mitochondrial 3-hydroxy-3-methylglutaryl-CoA synthase 2 (HMGCS2), while simultaneously enhancing ketone body utilization through upregulation of *β*-hydroxybutyrate dehydrogenase 1 (BDH1) and succinyl-CoA:3-ketoacid CoA transferase 1 (OXCT1). These coordinated effects may help preserve mitochondrial function, increase myocardial ATP production, reduce oxidative stress and apoptosis, and ultimately improve cardiac function in diabetic cardiomyopathy.

Similarly, studies involving *β*-hydroxybutyrate (*β*-OHB) administration and dapagliflozin treatment further support the concept of substrate reprogramming. Acute *β*-OHB infusion can increase myocardial ketone body oxidation while reducing fatty acid oxidation, suggesting that ketone bodies may directly influence cardiac substrate selection. Dapagliflozin has also been reported to increase hepatic ketogenesis and elevate circulating *β*-OHB levels, thereby facilitating a shift in cardiac mitochondrial metabolism from pyruvate oxidation toward ketone body oxidation. In heart failure models, dapagliflozin increased the relative oxidation rates of ketone bodies and fatty acids while suppressing pyruvate oxidation. These metabolic changes were associated with reduced myocardial oxidative stress and improved left ventricular ejection fraction, supporting the hypothesis that SGLT2 inhibitors may improve cardiac energetic efficiency by enhancing substrate flexibility and promoting the use of alternative fuels.

However, clinical evidence supporting this mechanism remains inconsistent. The EMPA-VISION trial—the first study to investigate energy metabolism in heart failure—demonstrated that following treatment with empagliflozin (10 mg/d for 3 months), patients with HFrEF or HFpEF showed no significant changes in cardiac energetics, as assessed by the phosphocreatine-to-ATP ratio, either resting or stress conditions, nor in serum metabolomics or ketone body levels (42).

Taken together, current evidence suggests that modulation of substrate flexibility, ketone body metabolism, and myocardial energy efficiency may represent a plausible pathway through which SGLT2 inhibitors exert cardioprotective effects. Nevertheless, this pathway remains incompletely validated in clinical settings and may vary according to disease phenotype, metabolic status, treatment duration, and methods used to assess cardiac energetics.

### 3.3 Vascular protection: endothelial homeostasis, angiogenic repair, and vascular remodeling

Endothelial dysfunction constitutes a critical pathological link in the pathogenesis and progression of cardiovascular diseases; its primary characteristics include reduced nitric oxide (NO) bioavailability, impaired endothelium-dependent vasodilation, heightened oxidative stress, activated inflammatory responses, and diminished vascular reparative capacity (43). Consequently, the improvement of endothelial homeostasis and vascular reparative capacity may represent one of the key mechanisms through which SGLT2 inhibitors exert their cardioprotective effects.

In models of ischemic disease, dapagliflozin has been shown to activate the PI3K–Akt–eNOS signaling pathway, thereby significantly enhancing endothelial cell tube formation, migration, and proliferation, and promoting angiogenesis following hindlimb ischemia (44). Additionally, the interaction with the pregnane X receptor (PXR) can enhance endothelial angiogenesis and alleviate cardiac insufficiency after myocardial infarction (45). This indicates that dapagliflozin may regulate the biological behavior of endothelial cells through multiple signaling pathways, promote angiogenesis, and improve the blood supply to ischemic tissues.

The homeostasis of endothelial function relies on the precise regulation of several signaling cascades, among which the interplay between Sirtuin 1 (SIRT1) and endothelial nitric oxide synthase (eNOS) is particularly pivotal (46). SIRT1, a key cellular regulator, plays a central role in metabolic and endocrine homeostasis as well as in maintaining endothelial integrity and overall cardiovascular function (47). In diabetic conditions, the level of SIRT1 in arteries is significantly reduced. Chronic supplementation with rmSIRT1 can improve endothelial function and vascular compliance and relieve endothelial and vascular dysfunction in diabetic mice by enhancing eNOS activity and inhibiting oxidative stress related to NADPH oxidase (NOX) (48). Dapagliflozin appears to activate the SIRT1 signaling pathway, promoting eNOS activation while suppressing reactive oxygen species (ROS) production, thus protecting endothelial cells from oxidative stress-induced premature senescence. Further studies have demonstrated that this protective effect is mediated through increased phosphorylation of eNOS at Ser1177 and reduced acetylation, both of which are regulated by SIRT1 (49). The study by Shi Tai et al. found that dapagliflozin can activate eNOS and reduce the production of ROS through SIRT1, prevent the impairment of endothelium—dependent vasodilation in a mouse model of type 2 diabetes, and improve arterial stiffness (50). In addition, both empagliflozin and dapagliflozin were shown to counteract TNF-α–induced endothelial dysfunction by decreasing ROS generation and restoring NO bioavailability (51).

Beyond these mechanisms, SGLT2 inhibitors also exert vasculoprotective effects through additional pathways. Notably, empagliflozin inhibited vascular smooth muscle cell (VSMC) proliferation and migration by downregulating the PDGF-R*β*/Akt/STAT3 pathway, thereby attenuating neointimal hyperplasia following carotid artery balloon injury—an effect observed independently of its glucose-lowering action (52). Furthermore, empagliflozin was reported to upregulate basic helix-loop-helix family member e40 (Bhlhe40), which suppresses the NOD-like receptor family, pyrin domain containing 3 (NLRP3) inflammasome and inhibits the osteogenic transdifferentiation of VSMCs, thereby delaying diabetes-associated vascular calcification (53).

Overall, SGLT2 inhibitors may confer cardiovascular benefits through multi-level vascular protective mechanisms: on the one hand, they can improve the SIRT1/eNOS/NO axis and redox homeostasis, thereby maintaining endothelial function and delaying endothelial senescence; on the other hand, they can promote post-ischemic angiogenic repair while inhibiting the abnormal proliferation, migration, and osteogenic transdifferentiation of vascular smooth muscle cells. Thus, vascular protection serves as a crucial mechanistic bridge linking the reprogramming of cardiovascular metabolism to the subsequent amelioration of oxidative stress, inflammatory responses, and myocardial remodeling.

### 3.4 Redox homeostasis: oxidative stress attenuation, ROS suppression, and antioxidant defense

Oxidative stress is a common pathological link in the pathogenesis and progression of cardiovascular diseases. In conditions such as diabetes, heart failure, ischemic heart disease, and atherosclerosis, metabolic disturbances, endothelial injury, and mitochondrial dysfunction collectively promote the excessive generation of ROS (54–56). ROS exhibit a typical “double-edged sword” effect. Under physiological conditions, low levels of transient and locally generated ROS can act as second messengers. Through H₂O₂-mediated reversible oxidation of protein cysteine residues, ROS regulate signaling pathways such as Mitogen-Activated Protein Kinase/Extracellular Signal-Regulated Kinase (MAPK/ERK), Phosphatidylinositol 3-Kinase/Protein Kinase B (PI3 K/Akt), Adenosine 5‘-monophosphate (AMP)-activated protein kinase (AMPK), and Ca^2^⁺-related pathways, and activate redox-sensitive transcription factors, including Nuclear Factor Erythroid 2-Related Factor 2 (Nrf2), Hypoxia-inducible factor 1-alpha (HIF-1*α*), Activator Protein-1 (AP-1), and Nuclear Factor kappa-B (NF-*κ*B). These processes contribute to cell proliferation, differentiation, immune defense, metabolic adaptation, and antioxidant stress responses (57–59). In contrast, when ROS production is excessive or antioxidant defense systems are insufficient, redox homeostasis is disrupted. Excessive ROS can induce DNA damage, lipid peroxidation, protein oxidation, and mitochondrial dysfunction. They further promote apoptosis, pyroptosis, necrosis, and ferroptosis through the cytochrome c/caspase-dependent apoptotic pathway, the Apoptosis signal-regulating kinase 1-c-Jun N-terminal kinase (ASK1–JNK)/p38 MAPK stress pathway, NF-*κ*B-mediated inflammatory responses, and NLRP3 inflammasome activation (60, 61). Therefore, restoring the balance between ROS generation and antioxidant scavenging may be one of the key cellular mechanisms through which SGLT2 inhibitors exert their cardioprotective effects.

SGLT2 inhibitors significantly alleviate oxidative stress-related injury by modulating multiple molecular targets involved in ROS production and clearance. For instance, a study by Xiaoling Li et al. demonstrated that empagliflozin reduced ROS production in human coronary artery endothelial cells (HCAECs) subjected to 10% cyclic stretch by inhibiting protein kinase C (PKC) activity and preventing NOX activation (62). Furthermore, in HCAECs exposed toTNF-α under flow conditions, it not only suppressed ROS generation but also restored NO bioavailability. These antioxidative effects were attributed to reduced intracellular Ca^2^⁺ influx and downregulation of sodium-hydrogen exchanger 1 (NHE1), which collectively contributed to decreased ROS levels under TNF-α stimulation (63).

Similarly, dapagliflozin has exhibited potent antioxidant activity. It was shown to reduce ROS levels in both rats and cardiac fibroblasts (CFs), thereby improving right ventricular structure and function in models of pulmonary hypertension (PH)-induced right heart failure (RHF) (64). Under high glucose conditions, dapagliflozin effectively reduced ROS and malondialdehyde (MDA) levels in CFs, suppressing fibrotic activity and improving DCM (65). In addition, both empagliflozin and dapagliflozin were found to attenuate oxidative stress by upregulating sirtuin 6 (SIRT6), inhibiting CF phenotypic transformation and proliferation, reducing ROS accumulation, and suppressing NOX4 expression (66).

In models of myocardial ischemia-reperfusion injury, dapagliflozin significantly attenuated oxidative stress by restoring glutathione peroxidase (GSH-Px) activity and reducing Malondialdehyde (MDA) levels. It also downregulated the expression of NOX2 and NOX4, thereby limiting ROS production. Moreover, it enhanced superoxide dismutase (SOD) activity and upregulated antioxidant transcription factors such as NRF2 and NAD(P)H:quinone oxidoreductase 1 **(**NQO1), indicating that its antioxidative effects may be mediated through activation of the NRF2/NQO1 pathway and inhibition of the NOX2/NOX4 axis (67).

Additionally, canagliflozin was shown to protect against palmitic acid (PA)-induced intracellular and lipid ROS accumulation, thereby mitigating cellular oxidative damage and delaying senescence (68).

Accordingly, by reducing ROS generation and enhancing antioxidant defense, SGLT2 inhibitors may create a more favorable intracellular environment for mitochondrial functional preservation, inflammatory suppression, and structural remodeling. In this sense, attenuation of oxidative stress provides a mechanistic transition from vascular protection to mitochondrial quality control.

### 3.5 Mitochondrial quality control: mitochondrial dynamics, mitophagy, and bioenergetic efficiency

Mitochondria are central organelles involved in myocardial energy metabolism, redox homeostasis, and cell fate regulation. Their structural integrity and functional stability are essential for maintaining normal cardiovascular physiology. However, mitochondrial dysfunction has been implicated in the pathogenesis of numerous CVD, including atherosclerosis, myocardial infarction, various cardiomyopathies, heart failure, hypertension, and arrhythmias (69). These mitochondrial defects often involve disruptions in mitochondrial dynamics (fission/fusion dysregulation) (70), impaired mitophagy (71), accumulation of reactive oxygen species (72), and reduced oxidative phosphorylation and ATP synthesis (73). In this context, the regulation of mitochondrial function represents an important cellular mechanism underlying the cardiovascular protection of SGLT2 inhibitors.

Mitochondrial morphology and function are maintained through a dynamic balance of fusion and fission processes, mediated by proteins such as mitofusin 2 (MFN2) and optic atrophy protein 1 (OPA1) for fusion, and dynamin-related protein 1 (DRP1) and fission protein 1 (FIS1) for fission. Empagliflozin has been shown to inhibit calcium-dependent ERK1/2 pathway activation, downregulate DRP1 and FIS1 activity, and thus reduce hyperglycemia-induced mitochondrial fragmentation and cardiomyocyte apoptosis (74). Canagliflozin, on the other hand, enhances mitochondrial fusion by activating the AMPK/KLF4 signaling pathway and upregulating MFN2 and OPA1 expression, which not only alleviates mitochondrial fragmentation in diabetic models but also induces adipose tissue browning to counteract metabolic disturbances (75).

Mitophagy, a selective form of autophagy targeting damaged mitochondria, is critical for mitochondrial quality control. PTEN-induced kinase 1 (PINK1) and Parkin RBR E3 ubiquitin ligase (PRKN) are central regulators of this process. Canagliflozin can inhibit isoproterenol (ISO)—induced excessive autophagy, restore mitochondrial quality by regulating the AMPK/PINK1/Parkin signaling pathway, and reverse abnormal mitochondrial structure in cardiac remodeling (76). Furthermore, in diabetic cardiomyopathy models, canagliflozin corrected hyperglycemia-induced impairments in PINK1/Parkin-dependent mitophagy, thereby improving mitochondrial function and cardiac performance (77). Empagliflozin has also demonstrated the ability to reverse STZ-induced reductions in state 3 respiration and mitochondrial membrane potential in diabetic rats. It enhances the expression of key regulators of mitochondrial biogenesis and respiration, including Peroxisome Proliferator-Activated Receptor Gamma Coactivator 1-alpha (PGC-1*α*), Nuclear Respiratory Factor 1 **(**NRF-1), Transcription Factor A (TFAM), DRP1, MFN1, and OPA1 (78). Empagliflozin has also been found to improve mitochondrial respiratory function and calcium cycling in a heart failure with preserved ejection fraction model by increasing the content of cardiolipin (a key lipid for maintaining the integrity of the respiratory chain), thereby alleviating diastolic dysfunction (79).

### 3.6 Immunometabolic modulation: inflammasome suppression, cytokine regulation, and macrophage polarization

Inflammation is extensively involved in the pathogenesis and progression of various cardiovascular diseases, serving as a critical pathological link connecting metabolic dysfunction, oxidative stress, mitochondrial damage, and myocardial remodeling. In conditions such as atherosclerosis, myocardial infarction, heart failure, and myocarditis, persistent low-grade inflammation can promote endothelial dysfunction, immune cell infiltration, cardiomyocyte injury, fibroblast activation, and extracellular matrix deposition. Clinical studies targeting inflammatory pathways have provided substantial support for the “inflammation hypothesis” in cardiovascular disease (80–83). Consequently, the suppression of inflammation may be regarded as a pivotal mechanistic layer—bridging the restoration of cellular homeostasis with the amelioration of structural remodeling—underlying the cardioprotective effects of SGLT2 inhibitors.

SGLT2 inhibitors significantly attenuate the expression of proinflammatory cytokines and adhesion molecules. In the *in vitro* study of dapagliflozin, it suppresses the activation of the NLRP3 inflammasome, thereby reducing the release of interleukin-1β (IL-1β), interleukin-6 (IL-6), and TNF-α, while also downregulating the expression of intercellular adhesion molecule-1 (ICAM-1) and vascular cell adhesion molecule-1 (VCAM-1). These effects mitigate myocardial and endothelial injury induced by lipopolysaccharide (LPS), TNF-α, or isoproterenol (ISO) (84). Similarly, empagliflozin has been shown in animal models to decrease TNF-α and monocyte chemoattractant protein-1 (MCP-1) levels and to inhibit activation of NF-*κ*B signaling pathway, thereby disrupting downstream inflammatory cascades (85).

Macrophages, as central regulators of inflammation, exhibit a dichotomous functional phenotype: the proinflammatory M1 phenotype exacerbates tissue injury, while the anti-inflammatory M2 phenotype promotes repair and resolution (86). SGLT2 inhibitors exert anti-inflammatory effects by modulating macrophage polarization. In an experimental autoimmune myocarditis (EAM) model, empagliflozin suppressed the expression of M1 macrophage marker inducible nitric oxide synthase (iNOS) while upregulating the M2 marker CD206, thereby alleviating myocardial inflammatory infiltration and fibrosis (87). Furthermore, both dapagliflozin and empagliflozin have been reported to directly suppress M1 macrophage inflammatory activity, reducing the secretion of key proinflammatory cytokines such as TNF-α, IL-1, and IL-6 (88).

By suppressing inflammatory signaling and limiting cytokine-driven fibroblast activation, SGLT2 inhibitors may ultimately attenuate fibrosis, hypertrophy, and adverse myocardial remodeling.

### 3.7 Myocardial remodeling as a convergent outcome: fibrosis inhibition, hypertrophy attenuation, and ventricular structural preservation

Myocardial remodeling represents a common pathological phenotype in the progression of various cardiovascular diseases and can be broadly classified into physiological and pathological remodeling. Physiological remodeling typically occurs during adaptive states—such as growth, exercise training, or pregnancy—whereas pathological remodeling is predominantly driven by factors including inflammation, ischemia, ischemia-reperfusion injury, biomechanical stress, neurohormonal overactivation, and increased afterload (89). Pathological myocardial remodeling is characterized by cardiomyocyte hypertrophy, cell death, fibroblast activation, collagen deposition, extracellular matrix remodeling, and abnormalities in ventricular geometry and function; moreover, it serves to drive the onset and progression of heart failure (89–91). Consequently, within the context of the cardioprotective effects exerted by SGLT2 inhibitors, myocardial remodeling constitutes a structural outcome resulting from the concerted action of various upstream mechanisms—including hemodynamic unloading, metabolic improvement, attenuation of oxidative stress, maintenance of mitochondrial function, and suppression of inflammation—rather than representing an isolated, singular mechanism.

Existing studies suggest that SGLT2 inhibitors can attenuate pathological cardiac hypertrophy and fibrosis across various experimental models. In models of cardiac remodeling induced by transverse aortic constriction (TAC) or isoproterenol, dapagliflozin has been shown to alleviate cardiac hypertrophy and interstitial fibrosis. The underlying mechanism may be linked to the upregulation of Acetaldehyde dehydrogenase2 (ALDH2): by modulating the NHE1/ROS/DNMT1 pathway, dapagliflozin reduces the methylation levels of the ALDH2 promoter and enhances the binding of (nuclear transcription factor Y subunit alpha)NFYA to the ALDH2 promoter, thereby ameliorating pathological changes associated with cardiac remodeling (92). This finding suggests that SGLT2 inhibitors may participate in the regulation of cardiac structural remodeling by influencing oxidative stress, epigenetic regulation, and metabolism-related protective factors.

Empagliflozin has also demonstrated the potential to directly modulate signaling pathways associated with cardiac hypertrophy. Studies have revealed that empagliflozin can negatively regulate the activity of various serine/threonine kinases in isolated diabetic hearts, with particularly pronounced reductions observed in the activity of RSK and Aurora A/B kinases. In models of cardiomyocyte hypertrophy, empagliflozin attenuates phenylephrine-induced cellular hypertrophy, suggesting a potential direct anti-hypertrophic effect on cardiomyocytes—an action that may be mediated by the inhibition of the RSK-NHE1 axis (93). Furthermore, by interacting with Frizzled (FZD) receptors, empagliflozin can inhibit Wnt/*β*-catenin/TCF7L2 signaling within cardiomyocytes; this mechanism serves to alleviate progressive TAC-induced cardiac hypertrophy and fibrosis, while also delaying the deterioration of cardiac function (94). These results suggest that the modulation of pathological hypertrophy by SGLT2 inhibitors may not be limited solely to hemodynamic unloading, but may also involve the direct regulation of pro-hypertrophic signaling pathways within cardiomyocytes.

In the context of metabolic stress-related myocardial injury, dapagliflozin similarly ameliorates pathological myocardial remodeling. Palmitate-induced lipotoxicity cell models demonstrate that dapagliflozin pretreatment attenuates cellular hypertrophy, fibrosis, and apoptosis in a concentration-dependent manner. In a high-fat diet-induced model of cardiac dysfunction, dapagliflozin reduces body weight and improves lipid profiles; furthermore, it negatively regulates the MAPK/AP-1 pathway—via an NHE1-dependent mechanism—thereby mitigating cardiac inflammation, myocardial hypertrophy, fibrosis, and pathological remodeling (95). This finding suggests that the cardioprotective effects of SGLT2 inhibitors against myocardial remodeling may be collectively attributed to the reduction of systemic metabolic burden, alleviation of lipotoxicity, suppression of inflammation, and attenuation of intracellular stress signaling within cardiomyocytes.

Overall, the improvement in myocardial remodeling conferred by SGLT2 inhibitors is not driven by a single pathway, but rather represents the convergence of multi-level protective effects. Hemodynamic rebalancing reduces ventricular wall stress and mechanical stretch stimuli; cardiovascular metabolic reprogramming attenuates glucotoxicity, lipotoxicity, and paracrine injury associated with epicardial fat; the restoration of redox homeostasis and mitochondrial quality control enhance cardiomyocyte energy supply and minimize cellular damage; and the suppression of inflammation mitigates cytokine-driven fibroblast activation and extracellular matrix deposition. Consequently, SGLT2 inhibitors may ultimately delay adverse myocardial remodeling and improve cardiac function by attenuating myocardial hypertrophy, fibrosis, apoptosis, and ventricular structural abnormalities.

### 3.8 From mechanistic networks to clinical heterogeneity: translational integration of SGLT2 inhibitor–mediated cardioprotection

**Figure 1:**
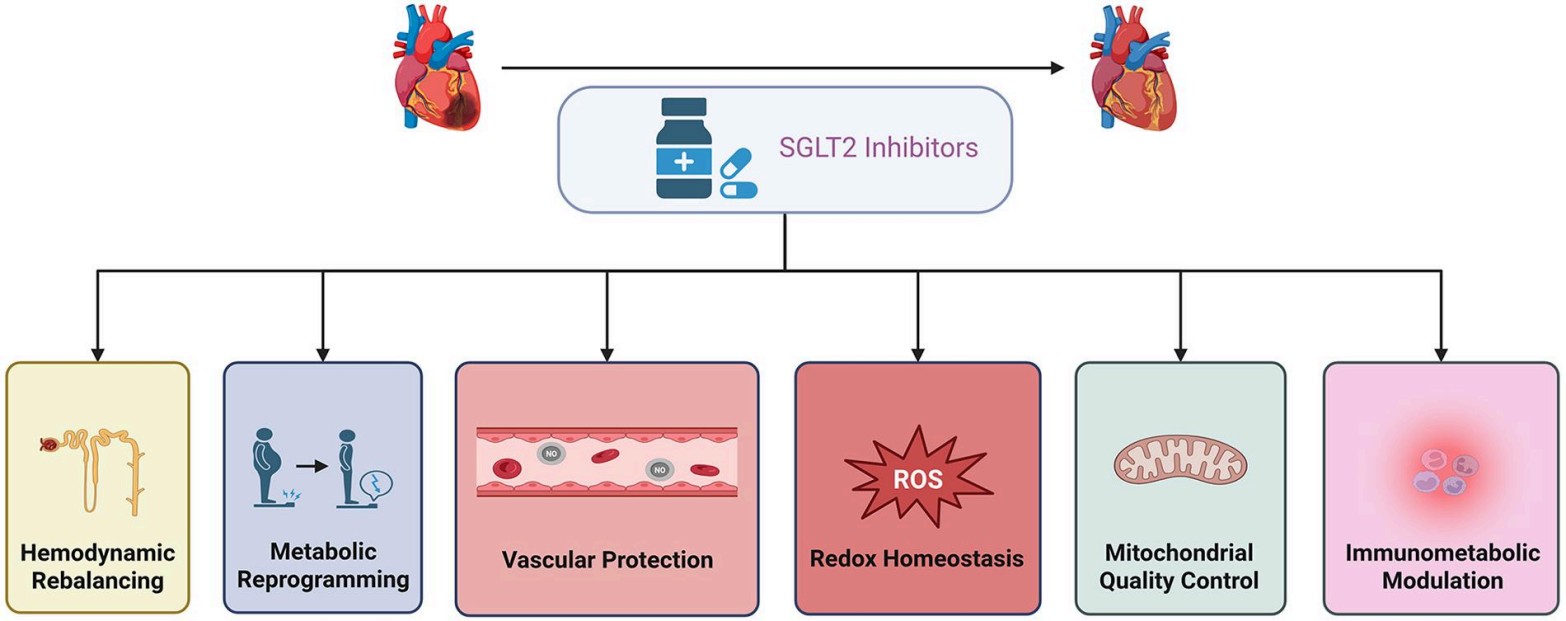
Mechanisms of SGLT2 inhibitor-mediated cardioprotection. Created in BioRender. https://BioRender.com/3oy4n38

Taken together, the cardiovascular protection conferred by SGLT2 inhibitors should not be attributed to a single dominant mechanism, but rather to a multilayered cardioprotective network involving systemic, metabolic, vascular, and cellular pathways (Figure 1). Within this network, hemodynamic rebalancing and cardiometabolic reprogramming may represent relatively upstream processes, reducing ventricular load, metabolic burden, epicardial adipose signaling, and energetic stress. These upstream adaptations subsequently converge on vascular protection, redox homeostasis, mitochondrial quality control, and immunometabolic modulation, ultimately contributing to the attenuation of fibrosis, hypertrophy, and adverse myocardial remodeling.

From a hierarchical perspective, the early benefits of SGLT2 inhibitors may be more closely related to hemodynamic rebalancing and cardiorenal regulation, whereas their long-term cardiovascular protection may depend on sustained metabolic adaptation, endothelial restoration, oxidative stress attenuation, mitochondrial preservation, and inflammatory suppression. This network-based interpretation may also help explain the clinical heterogeneity of SGLT2 inhibitor benefits across cardiovascular phenotypes. In patients with HFrEF, ventricular unloading, myocardial energy optimization, and attenuation of adverse remodeling may be particularly relevant. In HFpEF, reductions in body weight, epicardial adipose tissue, microvascular dysfunction, and systemic inflammation may contribute more prominently. For patients with ischemic heart disease or post-myocardial infarction: inhibition of oxidative stress, endothelial protection, and anti-fibrotic effects may be more relevant.

Nevertheless, the relative importance and causal hierarchy of these mechanisms remain incompletely defined. Much of the current mechanistic evidence is derived from cellular and animal models, and it remains uncertain whether different SGLT2 inhibitors exert distinct drug-specific effects across disease contexts. Moreover, the extent to which individual molecular pathways directly mediate clinical outcome improvements has not been fully established.

## 4 Clinical evidence for SGLT2 inhibitors in cardiovascular disease

Several landmark clinical trials have evaluated the effects of SGLT2 inhibitors in patients with heart failure, myocardial infarction, and other cardiovascular conditions. The study designs, patient populations, interventions, and major cardiovascular outcomes are summarized in Table 1.

**Table 1:** Clinical trials of SGLT2 inhibitors across the cardiovascular Spectrum.

Trial	SGLT2 inhibitor	Patients	Study design and sample size	Primary endpoint	Main findings	Clinical implication
DAPA-HF	Dapagliflozin 10 mg	Symptomatic chronic heart failure with reduced ejection fraction, LVEF ≤40%, with or without type 2 diabetes	Randomized, double-blind, placebo-controlled trial; *n* = 4,744	Composite of worsening heart failure or death from cardiovascular causes	Dapagliflozin significantly reduced the risk of worsening heart failure or cardiovascular death compared with placebo. The benefit was consistent regardless of diabetes status.	Established dapagliflozin as an effective therapy for HFrEF beyond glucose lowering.
EMPEROR-Reduced	Empagliflozin 10 mg	Symptomatic chronic HFrEF, LVEF ≤40%, with or without diabetes	Randomized, double-blind, placebo-controlled trial; *n* = 3,730	Composite of adjudicated cardiovascular death or hospitalization for heart failure	Empagliflozin reduced the combined risk of cardiovascular death or heart failure hospitalization, mainly driven by fewer heart failure hospitalizations. Renal outcomes were also improved.	Together with DAPA-HF, this trial supported the incorporation of SGLT2 inhibitors into foundational therapy for HFrEF.
EMPEROR-Preserved	Empagliflozin 10 mg	Symptomatic heart failure with preserved or mildly reduced ejection fraction, LVEF >40%	Randomized, double-blind, placebo-controlled trial; *n* = 5,988	Composite of adjudicated cardiovascular death or hospitalization for heart failure	Empagliflozin significantly reduced the composite endpoint, primarily by lowering heart failure hospitalization. The effect was observed irrespective of diabetes status.	Provided pivotal evidence for the use of SGLT2 inhibitors in HFpEF/HFmrEF, a population with historically limited disease-modifying therapies.
DELIVER	Dapagliflozin 10 mg	Symptomatic heart failure with mildly reduced or preserved ejection fraction, LVEF >40%	Randomized, double-blind, placebo-controlled trial; *n* = 6,263	Composite of worsening heart failure or cardiovascular death	Dapagliflozin reduced the risk of worsening heart failure or cardiovascular death in patients with LVEF >40%, with broadly consistent effects across prespecified subgroups.	Complemented EMPEROR-Preserved and strengthened the evidence for SGLT2 inhibitors across the full spectrum of heart failure ejection fraction.
EMMY	Empagliflozin 10 mg	Early post-acute myocardial infarction patients	Randomized, double-blind, placebo-controlled trial; *n* = 476; treatment initiated early after acute myocardial infarction	Change in NT-proBNP over 26 weeks	Empagliflozin was associated with a greater reduction in NT-proBNP over 26 weeks and favorable signals in cardiac structure and function. However, the trial was not powered for major clinical cardiovascular outcomes.	Suggested that early post-MI empagliflozin may improve neurohormonal and remodeling-related markers, but further outcome-driven evidence is required.
DAPA-MI	Dapagliflozin 10 mg	Acute myocardial infarction patients without prior diabetes or chronic heart failure, but with impaired left ventricular function or Q-wave MI	Randomized, controlled trial; *n* = 4,017	Hierarchical composite endpoint including death, heart failure hospitalization, recurrent MI, atrial fibrillation/flutter, new-onset type 2 diabetes, NYHA class, and weight reduction	Dapagliflozin improved cardiometabolic outcomes after acute MI, but did not show a clear reduction in conventional hard cardiovascular endpoints such as cardiovascular death, recurrent MI, or heart failure hospitalization.	Indicates potential metabolic and preventive benefits after MI, while its role in reducing post-MI hard cardiovascular events remains uncertain.
EMPACT-MI	Empagliflozin 10 mg	Acute myocardial infarction patients at increased risk of heart failure, including those with left ventricular dysfunction or congestion	Randomized, double-blind, placebo-controlled trial; *n* = 6,522	Composite of hospitalization for heart failure or death from any cause	Empagliflozin did not significantly reduce the primary composite endpoint of first heart failure hospitalization or all-cause death, although reductions in first and total heart failure hospitalizations were observed.	Does not support routine use of empagliflozin in all high-risk post-MI patients for mortality reduction, but suggests a possible role in reducing heart failure events.
DARE-AF	Dapagliflozin 10 mg	Patients undergoing first-time catheter ablation for persistent atrial fibrillation, without established indications for SGLT2 inhibitor therapy such as diabetes, heart failure, or CKD	Prospective, open-label, randomized trial; *n* = 200	Early recurrence or burden of atrial fibrillation after catheter ablation	A 3-month course of dapagliflozin did not significantly reduce early atrial fibrillation recurrence or atrial fibrillation burden after catheter ablation.	Does not support the routine use of dapagliflozin solely for preventing post-ablation atrial fibrillation recurrence in patients without conventional SGLT2 inhibitor indications.

### 4.1 Heart failure with reduced ejection fraction: established prognostic benefit and guideline-directed therapy

Heart failure with reduced ejection fraction (HFrEF) represents one of the cardiovascular phenotypes in which the clinical benefits of SGLT2 inhibitors are most firmly established. The progression of HFrEF is driven by impaired systolic function, neurohormonal activation, volume and pressure overload, metabolic dysfunction, inflammation, and adverse ventricular remodeling (96). Therefore, agents capable of modulating hemodynamic burden, cardiorenal-metabolic homeostasis, inflammatory signaling, and myocardial remodeling may provide multidimensional benefits in this population.

Large randomized controlled trials have shifted SGLT2 inhibitors from glucose-lowering agents to foundational therapies for HFrEF. The EMPEROR-Reduced trial, a multicenter, randomized, double-blind study, enrolled 3,730 patients with New York Heart Association (NYHA) class II–IV heart failure and a left ventricular ejection fraction ≤40%. The trial aimed to compare the therapeutic effects of empagliflozin (10 mg daily) vs. placebo. The results demonstrated that empagliflozin significantly reduced the incidence of the primary composite outcome of cardiovascular death or hospitalization for heart failure compared to placebo (hazard ratio [HR] = 0.75, 95% confidence interval [CI]: 0.65–0.86, *P* < 0.001), and the total number of hospitalizations due to heart failure was substantially lower in the empagliflozin group (HR = 0.70, 95% CI: 0.58–0.85, *P* < 0.001). In addition, Empagliflozin also markedly slowed the rate of decline in estimated glomerular filtration rate (eGFR), further supporting its renal protective role in patients with HFrEF (97).

Subsequent subgroup analyses confirmed the broad applicability of empagliflozin in HFrEF management. Its cardiovascular benefits were consistent regardless of baseline serum uric acid levels, NT-proBNP concentrations, diuretic use, or age (98–101). Moreover, empagliflozin reduced the risk of adverse cardiovascular and renal outcomes in patients with anemia (102) and significantly improved health-related quality of life in those with HFrEF (103).

It is noteworthy that the cardioprotective effects of empagliflozin are not solely attributable to its diuretic action (104). The mechanisms likely involve modulation of myocardial energy metabolism, cardio-renal axis preservation, and anti-inflammatory effects. Furthermore, when combined with mineralocorticoid receptor antagonists (MRAs), empagliflozin has been shown to further reduce heart failure-related morbidity and mortality (105).

The DAPA-HF trial further confirmed the therapeutic value of SGLT2 inhibitors in HFrEF. This phase 3, international, multicenter, randomized, double-blind, placebo-controlled trial enrolled 4,744 patients with NYHA functional class II–IV heart failure and an LVEF of ≤40%. The median follow-up duration was 18.2 months. Patients were randomly assigned to receive dapagliflozin 10 mg once daily or placebo in addition to recommended standard heart failure therapy. The primary endpoint was a composite of worsening heart failure or cardiovascular death. Worsening heart failure was defined as an unplanned hospitalization for heart failure or an urgent visit for heart failure requiring intravenous therapy. The results showed that dapagliflozin significantly reduced the risk of the primary composite endpoint: 386 of 2,373 patients (16.3%) in the dapagliflozin group and 502 of 2,371 patients (21.2%) in the placebo group experienced a primary endpoint event, corresponding to a hazard ratio of 0.74 (95% CI: 0.65–0.85; *P* < 0.001). This benefit was consistent regardless of whether patients had diabetes. Compared with placebo, dapagliflozin was associated with fewer total heart failure hospitalizations and cardiovascular deaths, as well as improvements in heart failure symptoms, functional status, and quality of life, which were also reflected by health status measures such as the The Kansas city cardiomyopathy questionnaire (KCCQ) (106).

Based on the evidence from large randomized controlled trials such as EMPEROR-Reduced and DAPA-HF, SGLT2 inhibitors have been included in the guideline-directed medical therapy (GDMT) for HFrEF. The 2022 AHA/ACC/HFSA guidelines for the management of heart failure clearly state that GDMT for HFrEF includes four basic classes of drugs: angiotensin receptor-neprilysin inhibitors (ARNI)/angiotensin-converting enzyme inhibitors (ACEI)/angiotensin receptor blockers (ARB), evidence-based beta blockers, mineralocorticoid receptor antagonists (MRA), and SGLT2 inhibitors. These drugs collectively constitute the current “four basic treatments” for HFrEF (107).

### 4.2 Heart failure with preserved ejection fraction: expanding therapeutic benefit in a historically unmet area

For a long time, there has been a lack of pharmacological treatment for HFpEF that can definitively reduce mortality or stably improve major hard endpoints; previous clinical trials for drugs such as RAAS inhibitors, aldosterone receptor antagonists, and ARNI have yielded generally limited results (108). However, the results of two large randomized controlled trials, EMPEROR-Preserved and DELIVER, have promoted the use of SGLT2 inhibitors in patients with heart failure and a left ventricular ejection fraction (LVEF) greater than 40%. Based on these data, the 2022 AHA/ACC/HFSA heart failure guideline gave SGLT2 inhibitors a Class IIa recommendation for patients with HFpEF (107), while the 2023 focused update of the ESC heart failure guideline further recommended dapagliflozin or empagliflozin for patients with Heart Failure with mildly reduced Ejection Fraction (HFmrEF) and HFpEF to reduce the risk of heart failure hospitalization or cardiovascular death, with a Class I, Level A recommendation (109).

The EMPEROR - Preserved study, a multicenter, randomized, double—blind trial of great significance, had a median follow—up period of 26.2 months, enrolled 5,988 patients with NYHA class II–IV heart failure and a left ventricular ejection fraction greater than 40%. Participants received standard-of-care therapy and were randomly assigned to receive empagliflozin (10 mg once daily) or placebo. The study found that empagliflozin significantly reduced the composite risk of cardiovascular death or hospitalization for heart failure, regardless of diabetic status. Specifically, among the 2,997 patients in the empagliflozin group, 415 (13.8%) experienced the primary outcome events (death from cardiovascular causes or hospitalization for heart failure), while among the 2,991 patients in the placebo group, 511 (17.1%) experienced the primary outcome events (6.9 vs. 8.7 events per 100 patient - years; hazard ratio, 0.79; 95% CI, 0.69 to 0.90; *P* < 0.001). In addition, empagliflozin also significantly reduced the total number of hospitalizations for heart failure (hazard ratio, 0.73; 95% CI, 0.61 to 0.88; *P* < 0.001) and slowed the decline eGFR compared to placebo, suggesting renoprotective effects (110).

Further analysis indicated that the cardiovascular benefits of empagliflozin were highly consistent across different clinical subgroups. Empagliflozin significantly reduced the risk of cardiovascular death or heart failure hospitalization regardless of diabetic status (111). It also lowered the frequency of emergency department visits and intensive care unit (ICU) admissions related to heart failure. Notably, these benefits were not associated with prolonged hospital stays, suggesting a sustained therapeutic effect throughout the disease progression (112).

Beyond reducing clinical events, empagliflozin significantly improved health-related quality of life (HRQoL) in patients with HFpEF, with benefits emerging early and persisting for at least one year (113). Moreover, empagliflozin demonstrated consistent efficacy across age groups, including outcomes such as cardiovascular death, initial heart failure hospitalization, and both first and recurrent hospitalizations (114).

Importantly, empagliflozin remained effective even in HFpEF patients with advanced chronic kidney disease, including those with an eGFR as low as 20 mL/min/1.73 m^2^. It reduced the incidence of acute kidney injury (AKI) and slowed the progression to macroalbuminuria across CKD and eGFR strata (113). Empagliflozin also produced early and sustained reductions in serum uric acid (SUA), offering additional clinical benefits by lowering the risk of hyperuricemia-related complications (115). Furthermore, its therapeutic efficacy was unaffected by baseline anemia, blood pressure, resting heart rate, or key biomarkers such as NT-proBNP and high-sensitivity cardiac troponin T (hs-cTnT) (116–119).

The DELIVER trial further confirmed the benefits of SGLT2 inhibitors in patients with heart failure and LVEF greater than 40%. DELIVER was a phase 3, international, multicenter, parallel-group, event-driven, double-blind, randomized controlled trial that enrolled 6,263 patients with chronic heart failure and an LVEF greater than 40%. The median follow-up duration was 2.3 years. Patients received dapagliflozin or placebo in addition to standard therapy. The results showed that dapagliflozin significantly reduced the risk of the primary composite endpoint of worsening heart failure or cardiovascular death. The primary endpoint occurred in 512 of 3,131 patients in the dapagliflozin group and in 610 of 3,132 patients in the placebo group, (hazard ratio, 0.82; 95% CI: 0.73–0.92; *P* < 0.001). This benefit was consistent in patients with and without diabetes and did not differ substantially between patients with LVEF ≥60% and those with LVEF <60% (120). Further analyses of DELIVER showed that dapagliflozin not only reduced the risk of first worsening heart failure or cardiovascular death, but also reduced total heart failure events and cardiovascular death, while improving symptom burden (120). A prespecified analysis showed that dapagliflozin reduced the risk of total heart failure events and cardiovascular death, and this benefit was consistent across different ranges of ejection fraction and multiple clinical subgroups (121).

These findings are consistent with those of EMPEROR-Preserved and indicate that the benefits of SGLT2 inhibitors extend across the spectrum of patients with heart failure and LVEF greater than 40%, primarily manifesting in reduced hospitalization for heart failure and worsening heart failure events, and are not affected by the state of diabetes.

### 4.3 Ischemic heart disease and post-myocardial infarction remodeling: cardiometabolic protection beyond glucose control

In recent years, the potential application of SGLT2 inhibitors in ischemic heart disease, particularly in patients after acute myocardial infarction, has attracted increasing attention. Following myocardial infarction, patients may develop myocardial necrosis, inflammatory activation, neurohormonal stimulation, ventricular remodeling, and an increased risk of heart failure (122). Given the established benefits of SGLT2 inhibitors in heart failure, whether early intervention with these agents after myocardial infarction can improve cardiac remodeling, reduce the risk of heart failure, and improve long-term prognosis has become an important focus of clinical research.

The EMMY trial was a multicenter, randomized, double-blind study that evaluated the efficacy and safety of empagliflozin initiated within 72 h after percutaneous coronary intervention in patients with large acute myocardial infarction. The study showed that, compared with placebo, empagliflozin significantly reduced NT-proBNP levels at 26 weeks. After adjustment for baseline NT-proBNP, sex, and diabetes status, NT-proBNP was reduced by 15%. This reduction was accompanied by significant improvements in echocardiographic parameters, including an increase in left ventricular ejection fraction, a decrease in mean E/e′, and reductions in left ventricular end-systolic and end-diastolic volumes. Because EMMY primarily focused on biomarkers and echocardiographic parameters, its findings are best interpreted as evidence that empagliflozin may improve post-infarction cardiac loading conditions, diastolic function, and remodeling-related surrogate endpoints, rather than as direct evidence of hard clinical outcome benefits (123).

The DAPA-MI trial further explored the role of dapagliflozin in patients after acute myocardial infarction with impaired left ventricular systolic function. This randomized, double-blind trial enrolled patients without prior diabetes or chronic heart failure. Dapagliflozin improved the primary hierarchical composite outcome, which included death, hospitalization for heart failure, nonfatal myocardial infarction, atrial fibrillation or flutter, new-onset type 2 diabetes, New York Heart Association functional class at the final visit, and body weight reduction of at least 5% at the final visit. However, this benefit was mainly driven by cardiometabolic outcomes, whereas dapagliflozin did not significantly reduce the composite endpoint of cardiovascular death or hospitalization for heart failure. Therefore, DAPA-MI supports the cardiometabolic benefits of dapagliflozin in patients after myocardial infarction, but does not establish a definitive reduction in post-infarction mortality or heart failure hospitalization (124).

The EMPACT-MI trial assessed empagliflozin in patients with acute myocardial infarction who were at increased risk of heart failure. Compared with placebo, empagliflozin did not significantly reduce the primary composite endpoint of first hospitalization for heart failure or death from any cause. Nevertheless, subsequent analyses suggested that empagliflozin reduced the risks of both first and total heart failure hospitalizations. These findings indicate that EMPACT-MI does not completely negate the potential value of SGLT2 inhibition after myocardial infarction; rather, it suggests that the benefit may be more concentrated on heart failure–related events than on short- to intermediate-term mortality reduction (125).

Overall, current evidence suggests that SGLT2 inhibitors have potential translational value in patients after myocardial infarction, particularly in reducing NT-proBNP levels, improving ventricular structural and functional parameters, optimizing cardiometabolic outcomes, and possibly reducing heart failure hospitalization. However, compared with the more established clinical benefits observed in HFrEF and HFpEF, the evidence for SGLT2 inhibitors after acute myocardial infarction remains more complex. EMMY mainly supports improvement in surrogate endpoints, the positive findings of DAPA-MI were largely driven by cardiometabolic outcomes, and EMPACT-MI did not meet its primary composite endpoint. These differences suggest that the benefits of SGLT2 inhibitors after myocardial infarction may depend on baseline risk, left ventricular functional status, the presence or absence of heart failure or diabetes, timing of treatment initiation, and endpoint selection. Further large-scale studies focusing on different myocardial infarction subtypes, heart failure risk strata, and long-term clinical endpoints are needed to clarify the optimal role of SGLT2 inhibitors in the management of ischemic heart disease and post-myocardial infarction care.

### 4.4 Arrhythmias: emerging evidence for atrial arrhythmia reduction and electrophysiological protection

In recent years, increasing evidence has suggested that SGLT2 inhibitors may be associated with a reduced risk of atrial arrhythmias, particularly atrial fibrillation (AF) and atrial flutter (AFL). AF is a common arrhythmia in patients with heart failure, diabetes, obesity, and chronic kidney disease, and its development is closely related to atrial structural remodeling, electrical remodeling, inflammation, oxidative stress, increased volume load, and metabolic dysfunction (126, 127). Given the integrated effects of SGLT2 inhibitors on hemodynamic regulation, metabolic reprogramming, oxidative stress attenuation, inflammation suppression, and myocardial remodeling, their potential impact on the development and recurrence of atrial arrhythmias has attracted growing attention.

Real-world pharmacovigilance data have provided early signals supporting this hypothesis. An analysis based on the US Food and Drug Administration Adverse Event Reporting System (FAERS) showed that, compared with other glucose-lowering drugs, SGLT2 inhibitor use was associated with a lower reporting rate of AF among patients with diabetes. This association remained generally unchanged after excluding reports in which insulin or antiarrhythmic drugs were listed as suspected or concomitant medications (128). However, because FAERS is a spontaneous reporting database, its findings are susceptible to reporting bias, indication bias, and residual confounding. Therefore, these results should be interpreted as an associative signal rather than direct evidence of a causal protective effect of SGLT2 inhibitors against AF.

Pooled analyses of randomized controlled trials have further supported the association between SGLT2 inhibition and reduced atrial arrhythmia risk. A meta-analysis including 33 placebo-controlled randomized controlled trials and 88,098 participants found that SGLT2 inhibitors significantly reduced the incidence of AF and the composite outcome of AF/AFL. Subgroup analyses suggested that this potential benefit may be more evident in patients with HFrEF, in men, with dapagliflozin treatment, and with longer follow-up durations of more than one year (129). These findings indicate that the effect of SGLT2 inhibitors on atrial arrhythmias may vary according to underlying disease phenotype, heart failure subtype, specific drug, and treatment duration.

A *post hoc* analysis of DECLARE-TIMI 58 provided important clinical evidence regarding dapagliflozin and AF/AFL risk. In this analysis of 17,160 patients with type 2 diabetes, dapagliflozin reduced the risk of first AF/AFL events by 19% and the total number of AF/AFL events by 23% during follow-up. This effect was generally consistent regardless of baseline history of AF, atherosclerotic cardiovascular disease, or heart failure (130). These results suggest that the potential atrial anti-arrhythmic effect of SGLT2 inhibitors may not be entirely dependent on pre-existing cardiovascular disease status, but may instead reflect their integrated regulation of metabolic burden, atrial pressure load, inflammation, and structural remodeling.

Beyond new-onset AF/AFL, the effect of SGLT2 inhibitors on AF recurrence, particularly after catheter ablation, has also been investigated. Some observational studies have suggested that, among patients with type 2 diabetes undergoing AF ablation, SGLT2 inhibitor use was associated with lower risks of cardioversion, new antiarrhythmic drug therapy, and repeat AF ablation, as well as lower incidences of heart failure exacerbation, all-cause hospitalization, and death (131). In addition, a prospective study by Harada et al. showed that, among patients with persistent AF and heart failure without type 2 diabetes, SGLT2 inhibitor treatment was associated with a lower AF recurrence rate and reduced left atrial pressure (132). These findings suggest that SGLT2 inhibitors may influence AF recurrence risk by reducing atrial pressure load, improving heart failure status, and attenuating atrial structural remodeling.

Nevertheless, evidence regarding the prevention of AF recurrence after catheter ablation remains inconsistent. The DARE-AF trial evaluated dapagliflozin for the prevention of early arrhythmia recurrence after AF catheter ablation showed that three months of dapagliflozin treatment did not reduce the risk of early post-ablation arrhythmia recurrence (133). This finding suggests that the effect of SGLT2 inhibitors on AF recurrence may depend on baseline metabolic status, heart failure status, post-ablation observation period, AF type, and endpoint definition.

Overall, current evidence suggests that SGLT2 inhibitors may reduce the risk of AF/AFL and may decrease AF recurrence or related medical interventions in selected patient populations. Potential mechanisms include reduced volume load, decreased left atrial pressure, improvements in body weight and metabolic inflammation, attenuation of oxidative stress, preservation of mitochondrial function, and suppression of atrial fibrosis and structural remodeling. However, compared with the established clinical benefits of SGLT2 inhibitors in HFrEF and HFpEF, evidence in the field of arrhythmias remains emerging and is mainly focused on AF/AFL. Ongoing or forthcoming studies, such as EMPA-AF and BEYOND, may help clarify whether SGLT2 inhibitors can serve as adjunctive therapeutic agents in the comprehensive management of atrial arrhythmias.

## 5 Safety profile and perioperative considerations of SGLT2 inhibitors

SGLT2 inhibitors are generally well tolerated, but several mechanism-related adverse events should be considered, including genital mycotic infections, urinary tract infections, diabetic ketoacidosis, osmotic diuresis–related volume depletion, hypotension, and transient changes in renal function (134–136). Rare but clinically serious events, such as Fournier's gangrene, severe urinary tract infections, lower-limb amputation in selected high-risk populations, and potential bone safety concerns, require individualized risk assessment, particularly in elderly, frail, or multimorbid patients (137). Therefore, the established cardiovascular, renal, and metabolic benefits of SGLT2 inhibitors should be balanced against patient-specific safety risks.

Perioperative use deserves particular attention. Although prior or preoperative exposure to SGLT2 inhibitors may be associated with a lower risk of postoperative acute kidney injury through mechanisms such as restoration of tubuloglomerular feedback, reduction of intraglomerular pressure, attenuation of tubular energy demand and renal hypoxia, and suppression of inflammation, oxidative stress, and fibrosis, the perioperative setting also increases susceptibility to euglycemic ketoacidosis/euglycemic diabetic ketoacidosis (eKA/eDKA) (138). Fasting, surgical stress, volume depletion, low-carbohydrate intake, and altered insulin requirements may promote lipolysis and ketogenesis, while normal or only mildly elevated glucose levels can delay recognition of eKA/eDKA (139). Accordingly, current ADA Standards of Care recommend holding SGLT2 inhibitors for 3–4 days before elective surgery and restarting them only after oral intake and hemodynamic stability have recovered and ketosis risk has resolved (140).

Clinical evidence regarding perioperative SGLT2 inhibitor safety remains mixed. In patients with T2DM undergoing metabolic and bariatric surgery, Chen et al. reported comparable 30-day AKI and ketoacidosis risks when SGLT2 inhibitors were discontinued at least 1 week before surgery, with a possible reduction in postoperative eGFR decline among patients with longer diabetes duration (141). In a large Veterans Affairs cohort, Tallarico et al. found a dual safety profile: preoperative long-term SGLT2 inhibitor use was associated with a modestly higher risk of postoperative eKA, but lower risks of perioperative AKI and 30-day mortality (142). Conversely, Dixit et al. found no significant adjusted increase in clinically diagnosed postoperative DKA among preoperative SGLT2 inhibitor users undergoing emergency surgery (143). Taken together, these findings suggest that perioperative SGLT2 inhibitor management should be individualized according to surgical urgency, fasting status, volume state, renal function, diabetes severity, and ketosis risk rather than applying a uniform risk assumption to all patients.

## 6 Conclusion

SGLT2 inhibitors have emerged as cardiovascular-metabolic therapies with benefits extending beyond glucose lowering. Their cardioprotective effects are best understood as an integrated network involving hemodynamic rebalancing, cardiometabolic reprogramming, vascular protection, redox homeostasis, mitochondrial quality control, immunometabolic modulation, and attenuation of adverse myocardial remodeling. This framework helps explain their established benefits in HFrEF, expanding role in HFpEF, and potential but less definitive effects in ischemic heart disease and atrial arrhythmias.

This review is strengthened by its phenotype-based clinical interpretation, and balanced discussion of safety and perioperative considerations. Nevertheless, important limitations remain. Many mechanistic findings are derived from preclinical studies, the relative contribution of different pathways likely varies across disease phenotypes, and evidence in post-myocardial infarction and arrhythmia populations remains less mature than that in heart failure. In addition, potential differences among individual SGLT2 inhibitors and the lack of direct mechanistic validation in human tissues require further investigation. Because this review is narrative and integrative in design, it did not apply a formal systematic review protocol, risk-of-bias assessment, or quantitative meta-analysis. Therefore, the interpretation of evidence may be influenced by study selection and the heterogeneity of available data. Future studies integrating clinical phenotyping, imaging, biomarkers, and multi-omics approaches are needed to clarify the causal links between molecular mechanisms and clinical outcomes and to support more precise use of SGLT2 inhibitors in cardiovascular disease.
